# ASO Author Reflections: Nationwide Data on Ostomy Use in Elderly Patients After Colorectal Cancer Surgery

**DOI:** 10.1245/s10434-021-10057-3

**Published:** 2021-05-06

**Authors:** Yu-Ting van Loon, Laurents P. S. Stassen, David D. E. Zimmerman

**Affiliations:** 1 Department of Surgery, Elisabeth-TweeSteden Hospital, Tilburg, The Netherlands; 2 Department of Surgery, Maastricht University Medical Center, Maastricht, The Netherlands

## Past

Up to 35% of Dutch elderly patients still receive an ostomy after colorectal cancer (CRC) surgery.^1^ The Dutch Total Mesorectal Excision trial showed a mortality rate of 57% in elderly patients, compared with 8% in younger patients once there is an anastomotic leakage.^2^ Eliminating this risk with its related morbidity and mortality could be important motives for both patients and surgeons to choose an ostomy in the elderly. Even though the impact of an ostomy on quality of life in the elderly after CRC surgery has been previously reported,^1^,^3^ little is known about its impact on their survival.

## Present

We performed a nationwide comprehensive analysis on the postoperative results and survival after CRC surgery in elderly Dutch patients (≥ 75 years), comparing the outcomes of patients with primary anastomosis (PA) with those with end ostomy (EO).^4^ Our analysis shows that elderly EO patients experience a significantly worsened 60-day, 90-day, and 3-year overall and relative survival, even after correcting for the available confounders using univariable, multivariable, and propensity score matching analyses.^4^ Based on these results, one might advocate that it is advisable to try to avoid the use of EO, regardless of comorbidities, age, or tumor stage. Various factors that may or may not be obvious, possibly detected at the initial outpatient assessment, might have led to the surgeons’ choice for an EO instead of an anastomosis.

## Future

The pace of population aging is much faster than in the past; elderly and frail patients are rapidly becoming the new challenges in the future of colorectal surgery. In contrary to popular belief, those elderly patients might not necessarily benefit from a stoma. More information is needed regarding how frailty combined with a stoma impacts postoperative outcomes and survival. Our hopes for the future are vested in the development of a surgical prediction model for colorectal surgery for elderly patients, focusing on which patients would benefit from conservative therapy and which patients would benefit from an ostomy. This is important for our clinical practice; it will change our surgical decision making and hopefully reduce the number of unnecessary elderly ostomy patients.
